# Revealing the influence of Cyano in Anchoring Groups of Organic Dyes on Adsorption Stability and Photovoltaic Properties for Dye-Sensitized Solar Cells

**DOI:** 10.1038/s41598-017-05408-8

**Published:** 2017-07-10

**Authors:** Wei-Chieh Chen, Santhanamoorthi Nachimuthu, Jyh-Chiang Jiang

**Affiliations:** Department of Chemical Engineering, National Taiwan University of Science and Technology, Taipei, 106 Taiwan, ROC

## Abstract

Determining an ideal adsorption configuration for a dye on the semiconductor surface is an important task in improving the overall efficiency of dye-sensitized solar cells. Here, we present a detailed investigation of different adsorption configurations of designed model dyes on TiO_2_ anatase (101) surface using first principles methods. Particularly, we aimed to investigate the influence of cyano group in the anchoring part of dye on its adsorption stability and the overall photovoltaic properties such as open circuit voltage, electron injection ability to the surface. Our results indicate that the inclusion of cyano group increases the stability of adsorption only when it adsorbs via CN with the surface and it decreases the photovoltaic properties when it does not involve in binding. In addition, we also considered full dyes based on the results of model dyes and investigated the different strength of acceptor abilities on stability and electron injection ability. Among the various adsorption configurations considered here, the bidentate bridging mode (A3) is more appropriate one which has higher electron injection ability, larger V_OC_ value and more importantly it has higher dye loading on the surface.

## Introduction

Recently, methods for improving photovoltaic properties of dye-sensitized solar cells (DSSCs) has attracted considerable interest because of their potential advantages towards light harvesting such as high power conversion efficiency, low cost, less environmental issues^1–5^. Among the major components of DSSC, dye sensitizer is indispensable one since it helps not only to absorb sunlight but also useful for electron generation and injection^6–8^. Originally, Ruthenium^9, 10^ based complexes and then porphyrin complexes^11–13^ have proposed as sensitizers and achieved photon to electron conversion efficiencies (PCE) greater than 10%. Over the two decades, the metal-free organic dyes have been proposed due to their several advantages over organometallic dyes^14–18^. Besides, recent achievement in the designing of organic dyes with a record efficiency over 14%^19^ has revealed a path for improving DSSCs performances.

To improve the efficiency of DSSCs, many research works have been directed all over the world, which includes the molecular designing of dyes holding desirable characteristics^3^ and finding the efficient counter electrode for the reduction of an electrolyte^20–24^. However, another key factor that controls the PCE is appropriate adsorption configuration for the dyes on the semiconductor surface. In a DSSC device, the excited electrons from the dye can efficiently inject to the surface only when dye molecules effectively bind to the surface. Previous studies reported that an inadequate binding of dye with TiO_2_ lowers the overall performance by 2%^25, 26^. Also, a recent study of Kley *et al*.^27^ on N3 dye explored the dependence of LUMO (lowest unoccupied molecular orbital) position on its binding modes. Hence, determination of the preferred adsorption mode of a dye is the most significant process in improving the efficiency of DSSCs.

It is well known that the anchor group plays a crucial role not only in binding the dyes with the oxide surface, but also affecting electron injection rate and packing of adsorbed dyes^28, 29^. The influence of different anchoring groups on the photovoltaic performance have demonstrated in earlier studies^30–33^. At present, carboxylic acid is the most used anchor groups because of its stability and easy synthesis. The previous experimental study indicates that Ru-based dyes with carboxylic acid as an anchoring group could adsorb on the surface five coordinated Ti atom via both in neutral and deprotonated forms^34^. Recently, Monti *et al*.^35^ found that the metal-free organic dyes also bind to the TiO_2_ surface similar to Ru-based dyes due to the same carboxylate (or carboxylic acid) in anchoring group moiety.

In addition, the substitution of cyano group together with a carboxylic acid in the anchoring part of organic dyes has been more popular in recent years^36–39^. In our earlier studies, we have also reported that the inclusion of cyano group in the anchoring part improved the optoelectronic properties of dyes^14, 15^. Furthermore, the different adsorption configurations of dyes containing carboxylic acid together with cyano group on the semiconductor surface have investigated, and it has been shown that the dye adsorption via cyanoacrylic group increased the adsorption stability compared to carboxylic acid^36, 40–43^. However, all those previous studies considered only limited adsorption configurations, and they mainly focused on the adsorption stability and geometries of the adsorbed dyes on the surface. Besides, the influence of this cyano group on the photovoltaic performances such as open circuit voltage (V_OC_), electron injection ability, and its effect on dye loading are still not clear. Hence, in the present study, we have designed the D-π-A type of dyes with COOH as an anchoring group and systematically investigated the effect of cyano group on all the possible adsorption configurations on the anatase TiO_2_ (101) surface and the photovoltaic properties using density functional theory methods. In addition, the impact of the designed dyes on the packing of adsorbed dye molecules on the oxide surface is investigated.

## Results and Discussion

Finding the preferred adsorption configuration of designed dyes on a TiO_2_ surface is a challenging task due to the larger system and conformational flexibility of dye molecules. Hence, in order to reduce the computational cost, initially, the effects of dye lengths on the adsorption properties have determined. For this, we have considered the dyes with different lengths such as An, A-An, π-A-An, and D-π-A-An, where D, π, A and An are a donor, π-bridge, acceptor and anchoring group, respectively. Based on our previous studies^14, 17^, we have designed a model dye which consists of 4-methoxy-N-(4-methoxyphenyl)-N-phenyl benzene amine (MPBA) as a donor, thiophene (T) as π-bridge, thienopyrazine (TP) as acceptor and COOH as an anchoring group. The benchmark calculations for the adsorption of dyes with different lengths were performed using PBE functional, and bidentate bridging configuration was used for the adsorption of dye on the TiO_2_ surface since the previous experimental studies of COOH anchoring group indicate that bidentate bridging mode is more stable^44–46^. The optimized geometries of model dye with different lengths adsorbed on TiO_2_ are shown in Figure S1 of supporting information. The adsorption energies are calculated using ${E}_{ads}={E}_{dye+surface}-({E}_{dye}+{E}_{Surface})$, and the values are listed in Table S1 of supporting information. The comparison of the calculated adsorption energies for different dye lengths shows that longer dye lengths are slightly (~0.04 eV) stable than the smaller dyes. Thus, these initial benchmark results indicate that even though the length of the dye is reduced, the adsorption energy value does not change significantly.

To get a deeper understanding of these dyes and surface interactions, we have also plotted the partial density of states (PDOS) for the two surface Ti atoms that are bonded with the adsorbed dyes, and it is shown in Figure S2 of supporting information. From this plot, we noticed a negative (upward) shift of Fermi level, while increasing the dye lengths, which is due to the increase in their HOMO levels with respect to the dye lengths. Further, the peak shape in the conduction band is significantly changed after the introduction of acceptor moiety along with anchoring group. However, it remains almost same for further substitutions. These above results indicate that the introduction of acceptor group is significant than the π-bridge and/or donor group. Hence, we assume that the model dye consisting of only acceptor and anchoring group (A-An) is sufficient to represent the real picture of the interactions of whole dye with the TiO_2_ surface.

**Figure 1:**
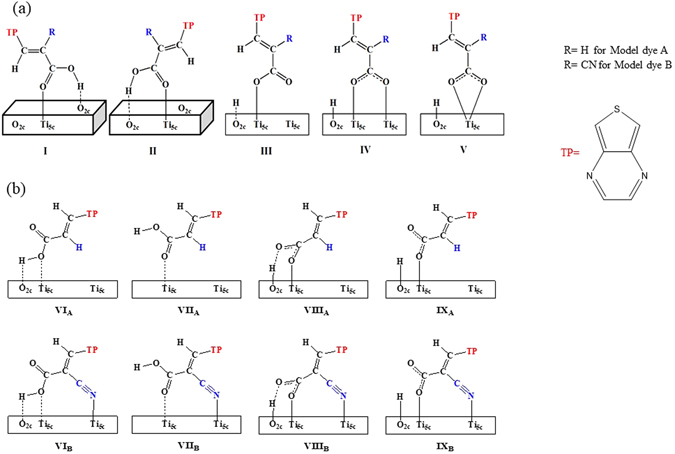
The schematic representation of considered adsorption configurations for model dyes A: TP-COOH and B: TP-^CN^COOH. ((**a**) configurations I-V for both dyes A and B adsorbed on surface via O-Ti bond and (**b**) configurations VI-IX for dye A adsorbed via O-Ti bond, and for dye B adsorbed via both O-Ti and N-Ti on TiO_2_).

To determine the suitable adsorption configurations, we have designed the model dyes consisting of only acceptor (TP) and anchoring group (COOH) moieties based on the above results. We also investigated the effect of the presence of cyano group on the adsorption configurations; hereafter we referred without and with cyano group dyes as A and B, respectively. Monodentate ester-type (MET), bidentate chelating (BC) and bidentate bridging (BB) configurations have considered for the adsorption of model dyes on the top layer of TiO_2_ anatase (101) surface which consists of five-coordinated titanium (Ti_5c_) and two-coordinated oxygen (O_2c_) atoms. For the adsorption configurations with deprotonated dyes (i.e. BB), the dissociated proton is migrated to the adjacent O_2c_ site of Ti_5c_ on the surface. The considered adsorption configurations for both model dyes A and B are illustrated in Fig. 1, and the calculated adsorption energies by PBE functional are summarized in Table S2 of Supporting information. As we can see from the Table S2, the bidentate chelating mode is relatively less stable, which is in agreement with the previous findings^47, 48^.

**Figure 2:**
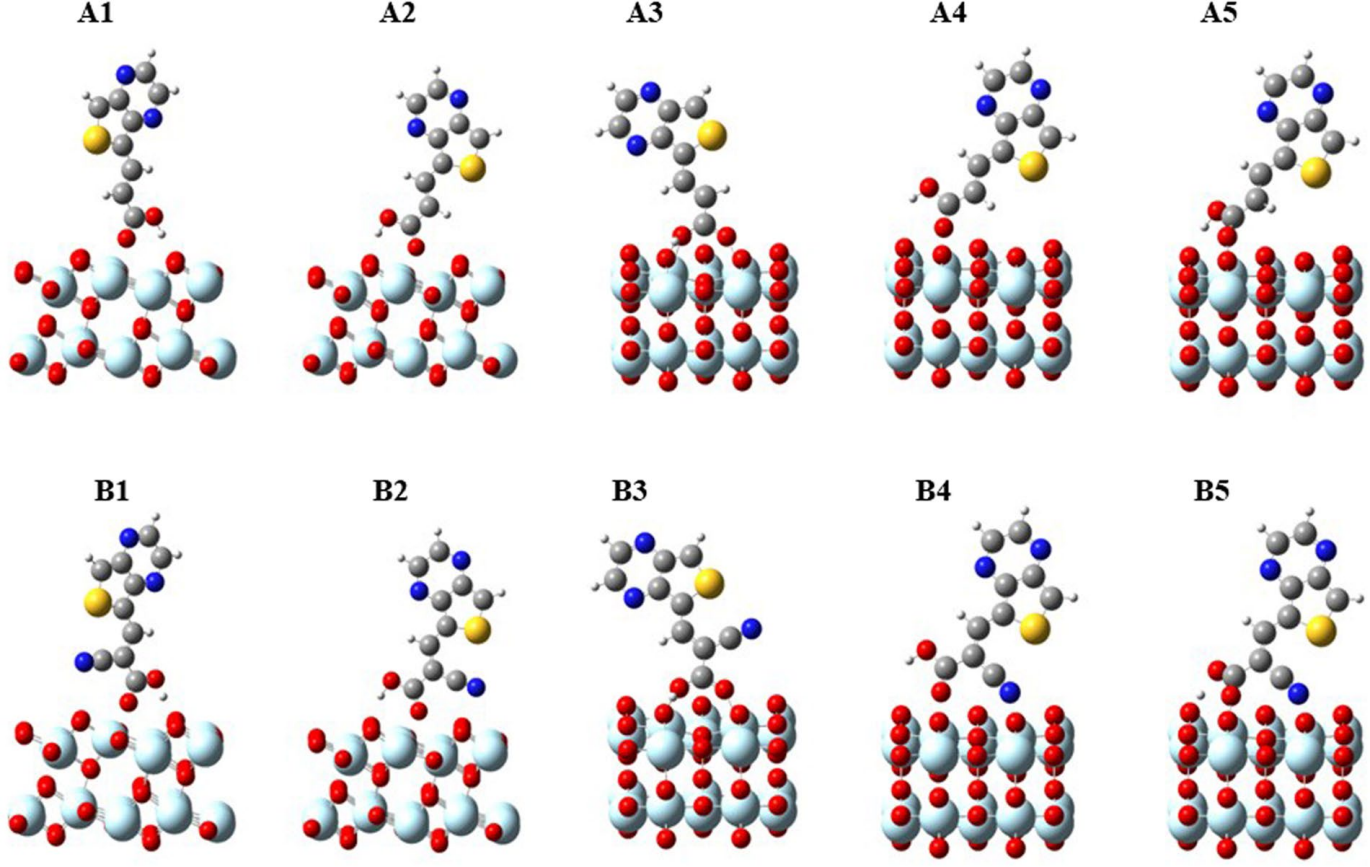
The optimized structures of most stable five adsorption configurations of model dyes (A and B) on TiO_2_.

Based on the above results, we have selected five most stable adsorption configurations I, II, IV, VII, and VIII in both A and B series (hereafter they are named as A1-A5 and B1-B5, respectively) and performed vdW corrected DFT calculations. The nature of each adsorption configuration has determined by vibrational frequency analysis, which confirms the minimum energy on the potential energy surface. The optimized adsorption configurations for both model dyes A and B are shown in Fig. 2 and the calculated adsorption energies, optimized geometrical parameters are listed in Table 1. We found that after vdW corrections, A2 and B5 are the most stable adsorption configurations, whereas A1 and B1 are the most stable in PBE calculation, which confirms the consideration of vdW is required for the interaction of dyes with metal oxide surfaces. Further, the calculated adsorption energies are higher than the previously reported values for dyes containing only carboxylic anchoring group and carboxylic with a cyano group^36, 40, 47^. The calculated adsorption energy for tridentate mode (B5) is −1.61 eV, which is higher than the recent study of Tsai *et al*.^40^, in which they reported that the tridentate adsorption mode has maximum adsorption energy of −1.31 eV. The larger adsorption energy in our results is due to the implementation of the high level of density functional with van der Walls interactions and higher threshold parameters in our calculations. We noticed that the presence of hydrogen bonds between the proton of carboxyl group and nearby surface O_2c_ atom leads to increase adsorption energies in A1, A2, and A5 configurations compared to A4. Also, we observed that the deprotonated BB configuration A3 is less stable than the other neutral dye configurations, even though it has shorter bond lengths between anchor and surface Ti_5c_ atoms (r_O-Ti_). Previously, Vittadini *et al*.^47^, and Mosconi *et al*.^49^, also reported higher stability of monodentate adsorption structures rather than bidentate bridging. To understand the effect of cyano group on the stability of adsorption, we compared both A and B series and found that, the substitution of cyano group has no significant influence on adsorption energy in the configurations which is not directly bound to the surface (B1-B3). Whereas it increases in B4 and B5 configurations because of its direct binding to the surface and among the considered adsorption configurations, B5 is the most stable one due to the strong hydrogen bonding along with two covalent bonds (O-Ti and N-Ti) with the surface, which is in agreement with the previous studies^41, 50^. The calculated bond lengths are r_O-Ti_ = 2.025 Å, r_N-Ti_ = 2.264 Å and r_H-O2c_ = 1.062 Å (See Table 1).

**Table 1:** The calculated adsorption energies E_ads_ (eV), selected bond lengths (r in Å) and bond distances (d in Å) for the five most stable adsorbed structures of model dyes without cyano (A series) and with cyano group (B series) on the TiO_2_ anatase (101) surface.

Parameters	Adsorption Configurations									
	A1	A2	A3	A4	A5	B1	B2	B3	B4	B5
E_ads_	−1.36	−1.41	−1.19	−1.15	−1.37	−1.36	−1.38	−1.16	−1.54	−1.61
r_O-Ti5c_	2.105	2.115	2.065, 2.043	2.159	2.085	2.137	2.159	2.080, 2.101	2.346	2.025
d_N…Ti5c_/r_N-Ti5c_	—	—	—	—	—	4.482	3.945	5.040	2.334	2.264
d_H…O2c_/r_H-O2c_	1.663	1.798	0.976	—	1.737	1.651	1.849	0.976	—	1.062
d_O…H_/ r_O-H_	1.015	1.005	—	0.983	1.009	1.021	1.007	—	0.983	1.500

Further, we also calculated the reconstruction energies ((E_Reconstruction_ = (E_SP_ − E_OPT_), where E_SP_ is the single point energy and E_OPT_ is the optimized energy) for the surface and model dye (π-A) with the configurations based on A2, A3, B3 and B5 to understand the smaller adsorption energies for A3 & B3 configurations and the calculated values are given in Table 2. It has been observed that the dye reconstruction energies are lower than the surface reconstruction energies for all the cases. The surface reconstruction energy for A2 is the smallest one compared to others, which indicate that the neutral form of dye does not influence the surface structure a lot when it adsorbed. Whereas, the surface reconstruction energy values are significantly larger in the deprotonated form of dyes (A3, B3, and B5). Importantly, the calculated reconstruction energies for A3 & B3 are greater than B5, which shows that the smaller adsorption energy for these configurations is mainly due to the surface reconstruction not due to the weaker interaction between the dye and TiO_2_ surface. To confirm the stability of these configurations, we have also calculated the interaction energy using reconstruction energies for both surface and dyes and calculated adsorption energies $({E}_{int}={E}_{ads}-({E}_{Reconstruction}^{Surface}+{E}_{Reconstruction}^{Dye}))$. The calculated values are listed in Table 2, which shows that even though configuration A3 has low adsorption energy, it is having larger interaction energy than B5 and it confirms that A3 adsorbed more stably on the surface than the other configurations.

**Table 2:** The calculated reconstruction energies (in eV) for both dye and surface, and interaction energy of dye with the surface (*E*_int_ in eV) for selected adsorption configurations of model dyes.

Parameters	Configurations			
	A2	A3	B3	B5
${E}_{\mathrm{Re}\,construction}^{Surface}$	0.26	1.51	1.44	1.00
${E}_{\mathrm{Re}\,construction}^{Dye}$	0.10	0.28	0.22	0.12
*E*_int_	−1.77	−2.98	−2.82	−2.73

**Figure 3:**
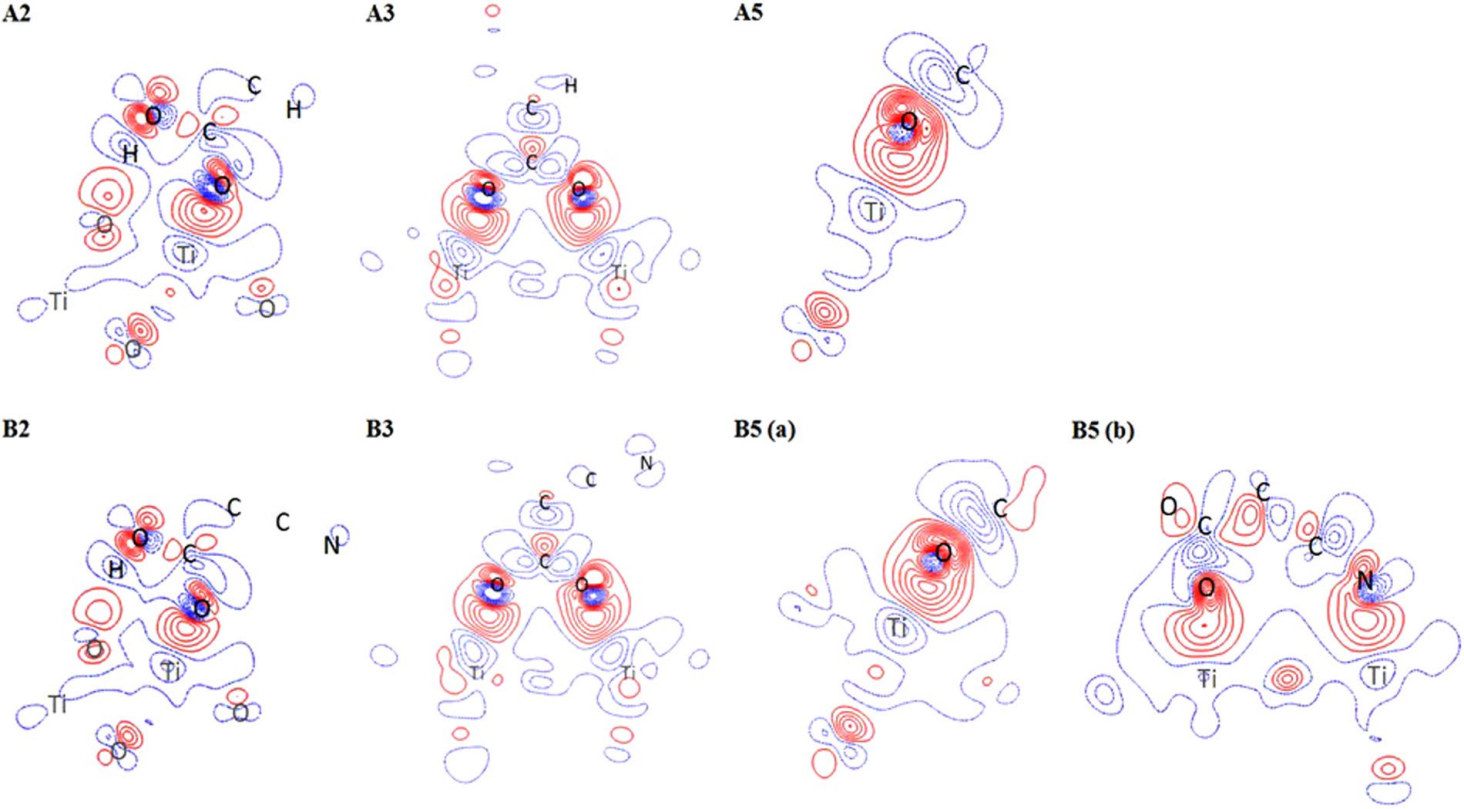
The electron density difference plot of configurations 2, 3, and 5 for both TP-COOH (A) and TP-^CN^COOH (B) dyes.

To deeply understand the interaction between dye and surface, the electron density difference (EDD) has obtained by cleaving the plane along the anchoring group and the attached surface atoms. An EDD diagram is a representation of the changes in electron density before and after the dye adsorption on the surface and is calculated as ${{\rm{\Delta }}}_{\rho }={\rho }_{dye+surface}-({\rho }_{dye}+{\rho }_{surface})$. The obtained EDD plots for configurations 2, 3, and 5 of both A & B series are shown in Fig. 3. An increase in electron density between the anchor O and surface Ti_5c_ atoms has been observed in A2, B2, A3 and B3 configurations. The gain in electrons are more in A2 and A3 configurations than B2 and B3, respectively, which leads the shortening of O-Ti bonds (r_O-Ti5c_) by 0.044 Å in A2 and by 0.036 Å in A3 than B2 and B3 configurations, respectively. These results indicate that the substitution of cyano group on the anchoring moiety (B2 and B3) does not improve the electronic interaction between dye and surface. In order to understand the adsorption via cyano group, we obtained EDD plot by cleaving the plane which contains C, O, Ti atoms and another plane contain C, N, Ti atoms for B5 configuration and are shown in Fig. 3 as B5(a) and (b), respectively. Figure 3B5(b) shows the additional contours along anchor N and surface Ti_5c_ atoms, which ensures the existence of interaction via the Ti-N bond. Our calculations indicate that the substitution of cyano group helps to increase the stability only when it binds to the surface. In addition, the EDD plot shows the electron density contours between anchoring group and two surface Ti atoms in A3 & B3 are even denser than B5 configuration; this again confirms that the less stable in adsorption energy of a bidentate bridging configuration is not due to the weaker interaction between dye and surface.

The overall efficiency, PCE of solar cell devices can be expressed as^51^,

1 $$PCE=\frac{{J}_{SC}{V}_{OC}FF}{{P}_{inc}}$$

where *J*_*SC*_ is the short circuit current density, *V*_*OC*_ is the open circuit voltage, FF is the cell fill factor and *P*_*inc*_ is associated with the incident light power which is a constant. In DSSCs, *V*_*OC*_ is related to the difference between quasi-Fermi level of TiO_2_ $({\varepsilon }_{F})$ and the electrochemical potential of redox couple of electrolytes $({\mu }_{Redox})$ and defined as^52, 53^

2 $$q{V}_{OC}={\varepsilon }_{F}-{\mu }_{Redox}$$

**Figure 4:**
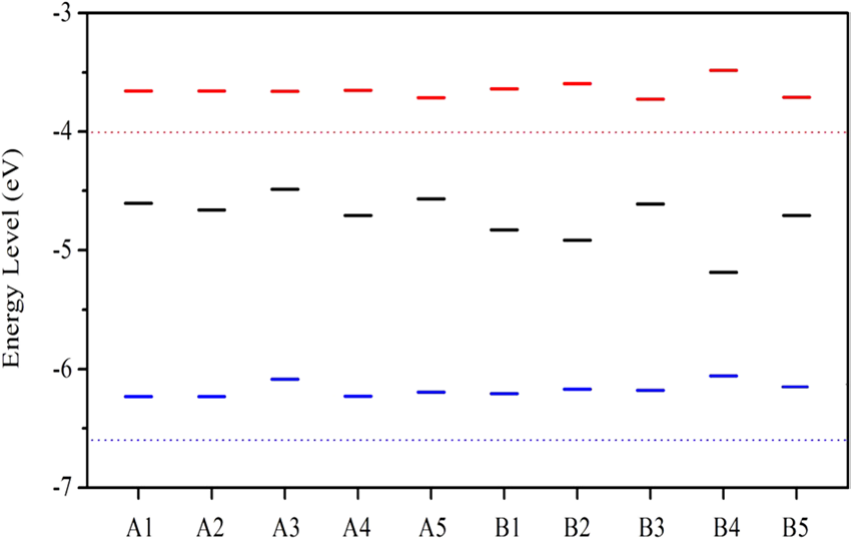
The calculated energy levels (in eV) including conduction band (red), Fermi level (black) and valence band (blue) of most stable five configurations for both dyes A and B. The dashed lines represent the pristine TiO_2_.

**Figure 5:**
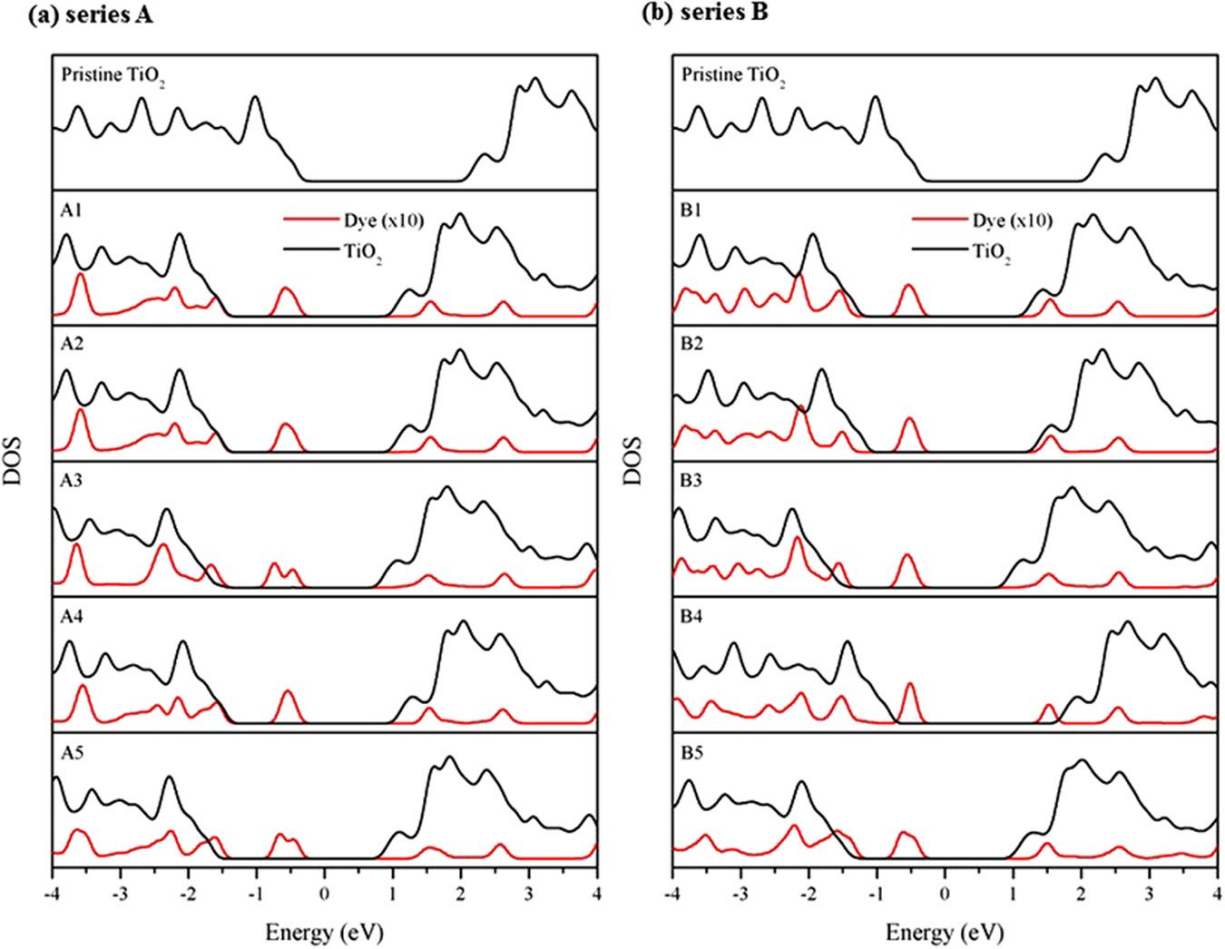
The projected density of states of TiO_2_ and dyes of five stable configurations of both A and B dyes. (The black lines represent DOS of the TiO_2_ whereas the red lines denote either TP-COOH or TP-^CN^COOH in dye molecule with enlarged 10 times of y-axis.)

The change in *V*_*OC*_ is associated with the shift in the Fermi energy levels of the TiO_2_ after the adsorption of dyes^54^. Thus, the *V*_*OC*_ for different adsorption configurations of designed model dyes is calculated using the energy level diagram. From Fig. 4, it is seen that the Fermi levels of A1, A2, and A4 configurations remain same, which indicate that the hydrogen bonding in those configurations does not influence the energy levels. Whereas, the deprotonation of dye in the adsorption configuration 3 and 5 has increased the Fermi level significantly in both the series. The projected DOS of pristine TiO_2_ and each dye configurations are shown in Fig. 5. The Fermi levels of studied systems are corrected to zero. As can be seen from Fig. 5, the deprotonated dye adsorption configurations 3 and 5 for both A and B dyes shift the Fermi level towards the conduction band. This Fermi level shift is larger in A3 and B5 configurations, which leads to smaller band gap in those configurations compared to bare TiO_2_. The calculated bandgap for the different dye adsorption configurations is listed in Table 3. Furthermore, the Fermi level of A3 configuration is more closer to conduction band than the other configurations, hence, we expect that A3 configuration have higher *V*_*OC*_ values than the other configurations, which is in agreement with the recent report by Suresh *et al*.^55^, which showed anionic dye has enhanced *V*_*OC*_ than neutral dye.

**Table 3:** The calculated bandgap (in eV) of TiO_2_ after the adsorption of dyes with studied configurations.

System	1	2	3	4	5
A	2.574	2.574	2.425	2.578	2.479
B	2.569	2.575	2.452	2.571	2.421
Pristine TiO_2_	2.593				

**Figure 6:**
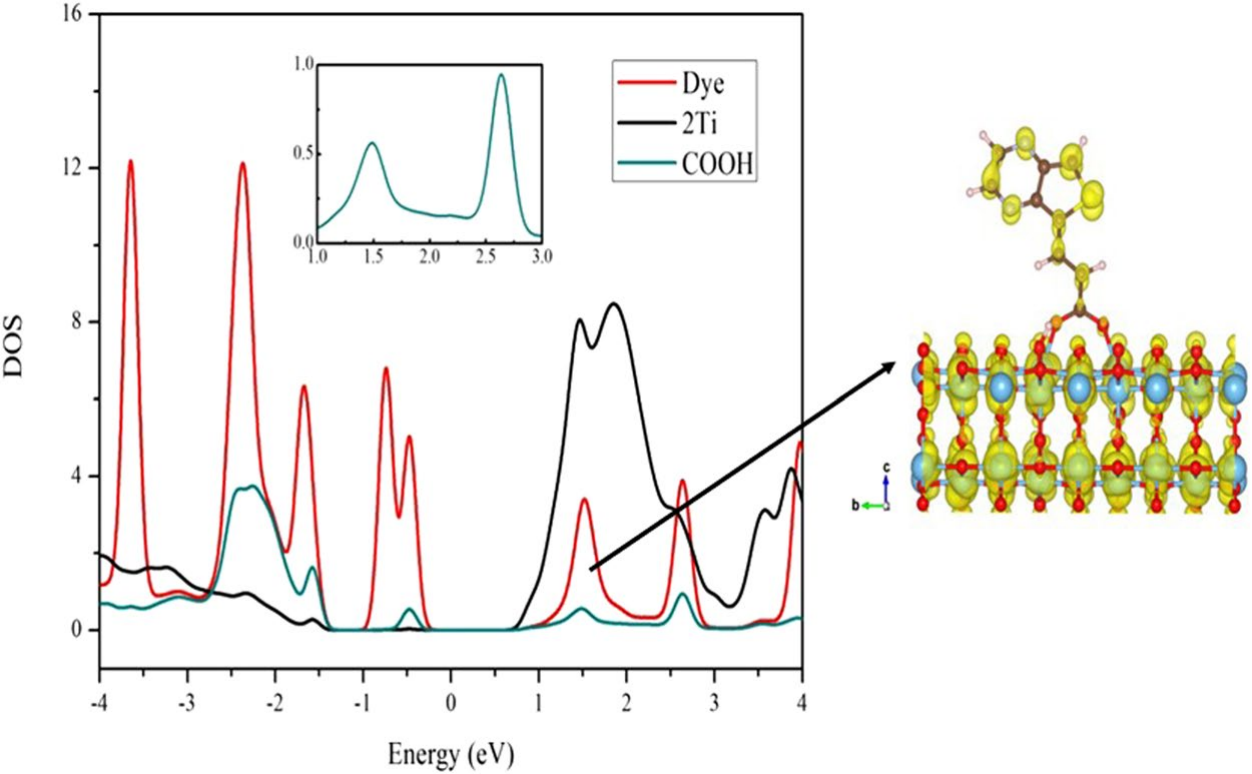
The DOS plot for the model dye adsorption configuration A3 along with its partial charge density for the band corresponds to long wavelength absorption band; isovalue 0.0004 a.u.

**Figure 7:**
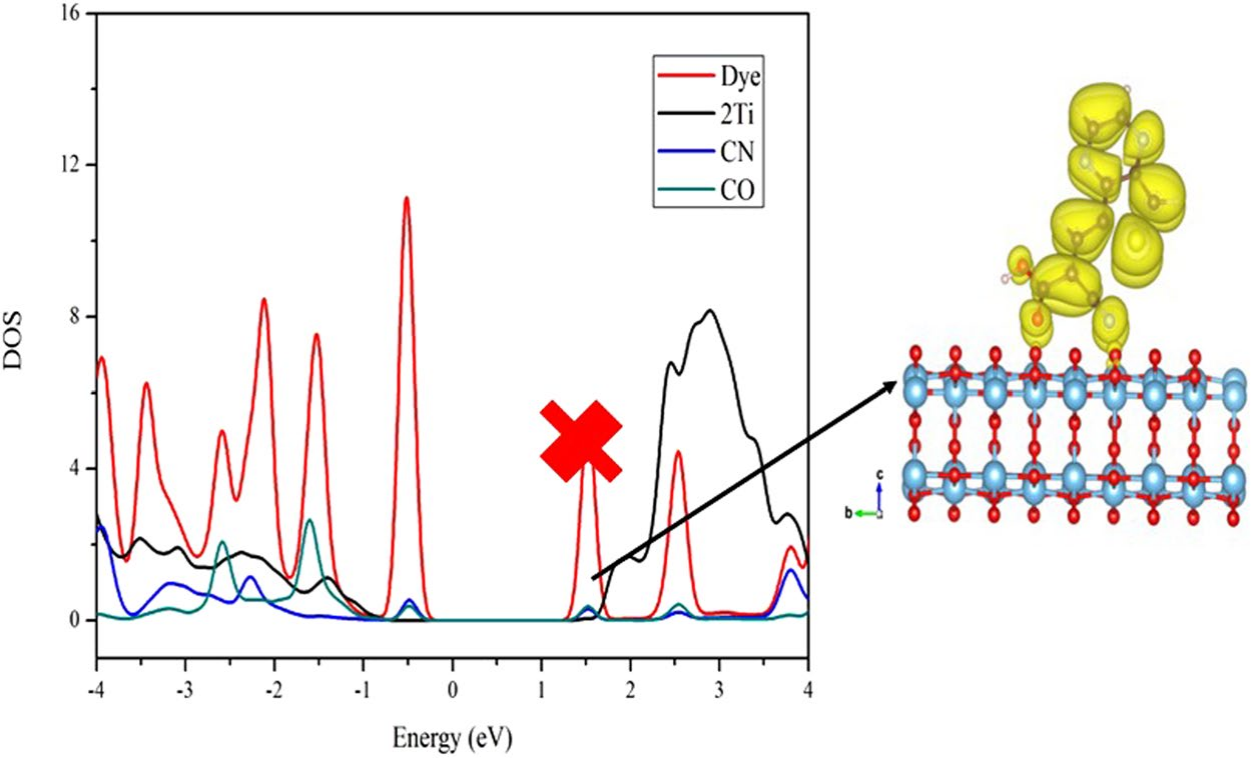
The DOS plot for the model dye adsorption configuration B4 along with its partial charge density for the band corresponds to long wavelength absorption band; isovalue 0.0004 a.u.

The electron injection ability can be examined by calculating the overlap between the anchoring group moiety (COOH) and the surface Ti atoms. The more overlap between these two regions will increase the electron injection, which leads to higher J_SC_ and thereby increase in overall PCE values since J_SC_ strongly depend on the electron injection efficiency (Φ_inject._) of the dyes^56^. To know the electron injection efficiency of each configuration, the overlap between the anchoring group and surface binding atoms (Ti) was considered from 0 to 3 eV in the DOS plot, and we integrate this overlap area for different dye adsorption configurations which are listed in Table 4. Among all, the adsorption configuration A3 possesses larger overlap integral, which may have higher J_SC_ value compared to others. Besides, we observed that the overlap integral values of dye with configurations A1-A4 are larger than B1-B4. The presence of hydrogen bonding enhances the overlap in A1 configuration than A4, and also the formation of the additional Ti-N bond increases the overlap in B5 than A5. However, the overlap in B5 is still lower than the A3 and B3 configurations. These results indicate that the substitution of cyano group in the anchoring part does not improve the electron injection ability, even it directly binds to the surface. To better account for the role of cyano group on the electron injection ability, we plotted PDOS for the different dye adsorption configurations. The band decomposed partial charge density states is also selected to display the electron distribution of the respective bands in the CB of the dye/TiO_2_ system. The PDOS plot along with band decomposed partial charge density for A3, and B4 configurations are presented in Figs 6 and 7, respectively. The same for remaining configurations (A2, B2, B3 & B5) are given in Figures S3–S6 of supporting information, respectively. Figure 6, S3–S5 clearly show that the electrons are localized in acceptor and anchoring moieties for A2, A3, B2, and B3 configurations. However, the presence of cyano group in B2, B3, and most stable B5 configurations decreases the peak intensity of COOH (inset of Figures S4, S5 and S6), which might be due to the strong withdrawing ability of cyano group than COOH. Further, we found that the peak corresponds to long wavelength absorption band for B4 is not overlapped with surface Ti atoms (see Fig. 7). Thus electron injection through this configuration is comparatively less even though the electrons are located in the anchoring group moiety. The above results show that the presence of cyano group in the anchoring group decreases the electron injection ability when it does not involve in the binding with surface atoms. On the other hand, configurations in which cyano group binds with surface atoms along with carboxyl group did not improve the electron injection ability. Hence, the present study reveals that the presence of cyano group in the anchoring part only helps to increase the stability of the adsorption, when it binds to surface and not in the improving the photovoltaic properties.

**Table 4:** The integral area obtained from the overlap between anchoring group and two surface Ti atoms for the studied dye adsorption configurations.

Dyes	1	2	3	4	5
A	0.434	0.432	0.589	0.353	0.369
B	0.304	0.294	0.443	0.201	0.434

**Figure 8:**
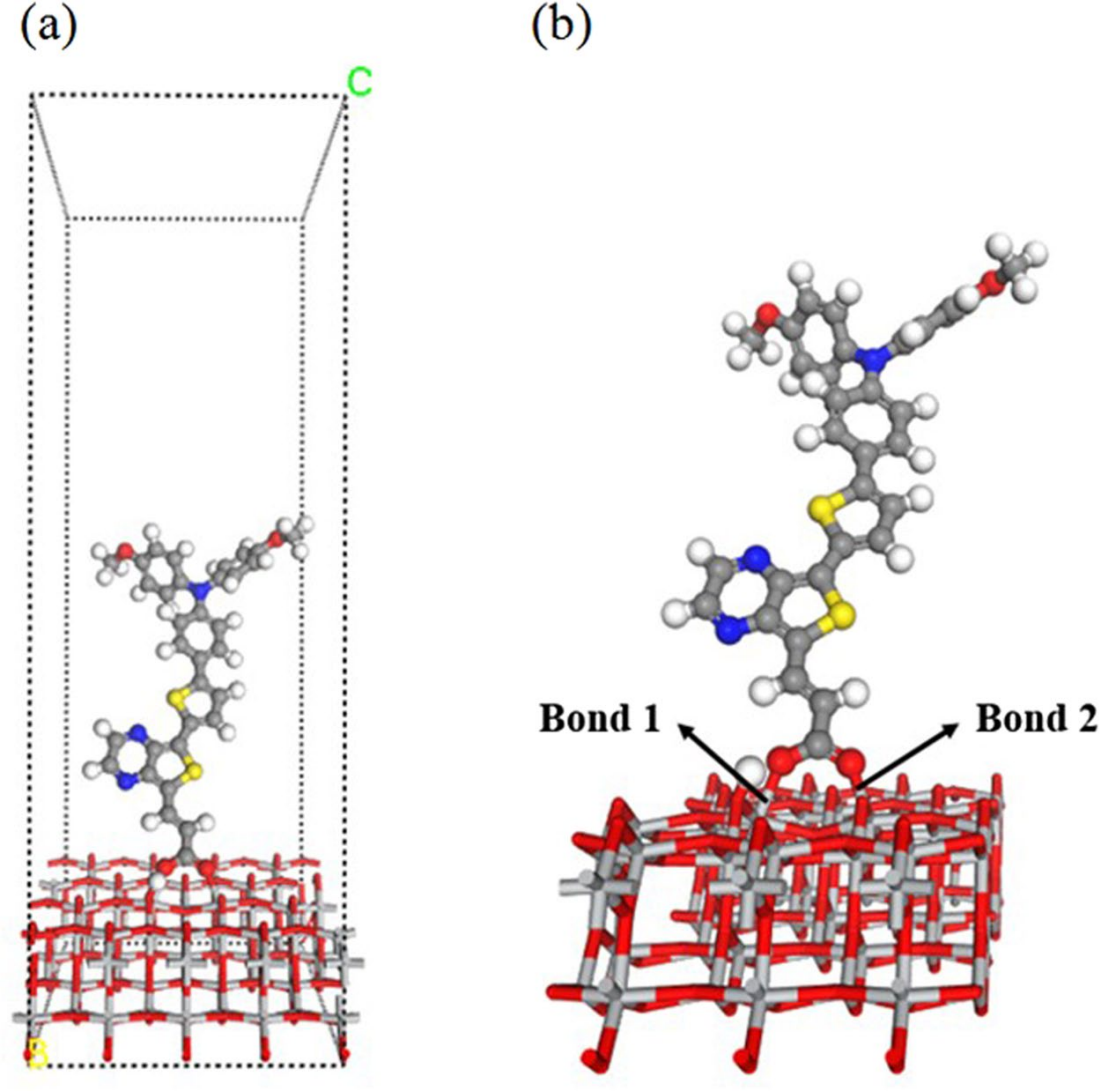
The optimized geometry of designed full dye (Dπ-TP-COOH) adsorbed onto TiO_2_ anatase (101) surface with the bidentate bridging mode.

To deeply understand the interaction of dyes with an oxide surface, we considered the adsorption of dye with D-π-A configurations on TiO_2_ (101) surface. In our earlier studies^16, 17, 57^, we found that the strength of acceptor moieties affects the optoelectronic properties, therefore here we aimed to explore the effect of the different withdrawing ability of acceptor moieties on the electron injection process. For this, we designed dyes with three different strength of acceptors such as dicyanomethylidene-cyclopentadithiophene (CDM), cyclopentadithiophene (CDT), and thienopyrazine (TP) along with MPBA as a donor, thiophene as π-bridge, and COOH (with and without cyano) as an anchoring group. Thus, totally six D-π-A type dyes (Dπ-CDM-COOH, Dπ-^CN^CDM-COOH, Dπ-TP- COOH, Dπ-^CN^TP-COOH, Dπ-CDT-COOH and Dπ-^CN^CDT-COOH) have designed and investigated their adsorption on TiO_2_ (101) surface using van der Walls corrected DFT method. Bidentate bridging mode (BB) was considered for the adsorption of full dyes since this adsorption configuration possesses most desirable characteristics based on our previous model dyes calculations. The optimized structure of Dπ-TP-COOH dye adsorbed on TiO_2_ is shown in Fig. 8 as an illustrative of other systems. The calculated adsorption energy values for all the designed full dyes are listed in Table 5. The calculated adsorption energy values for both model and full dyes indicate that, even though the different acceptors substituted, the calculated adsorption energy values for full dyes are analogous to that of our model dyes, which validate our above model dyes calculations. We also found that the substitution of cyano group increases adsorption energy very slightly in the designed dyes.

**Table 5:** The calculated adsorption energies (E_ads_ in eV)) for the considered full dyes on TiO_2_ anatase (101) surface.

System	E_ads_(eV)
Dπ-CDM-COOH	−1.16 (−0.42)
Dπ-^CN^CDM-COOH	−1.19 (−0.39)
Dπ-TP-COOH	−1.18 (−0.48)
Dπ-^CN^TP COOH	−1.28 (−0.47)
Dπ-CDT-COOH	−1.20 (−0.49)
Dπ-^CN^CDT-COOH	−1.24 (−0.45)

The values in the parenthesis are adsorption energies before considering vdW correction.

**Figure 9:**
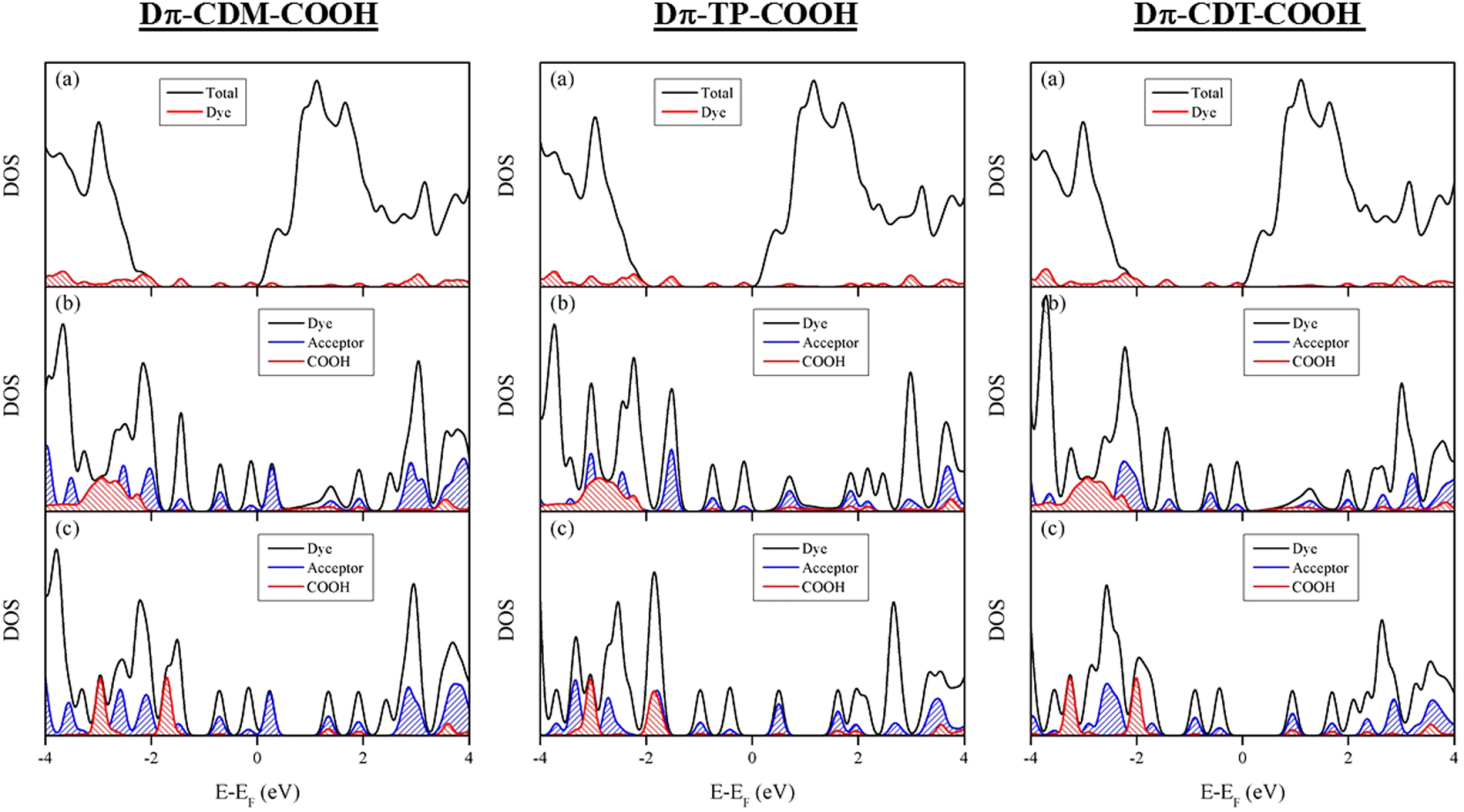
(**a**) Total density of states (black line) of dye with surface and that projected on dye molecules (red line), (**b**) Total density of states (black line) of dye and that projected on acceptor (Blue line) and COOH (red line) groups after the adsorption and (**c**) Total density of states (black line) of dye and that projected on acceptor (Blue line) and COOH (red line) groups before the adsorption of different acceptor substituted dyes on TiO_2_ surface.

**Figure 10:**
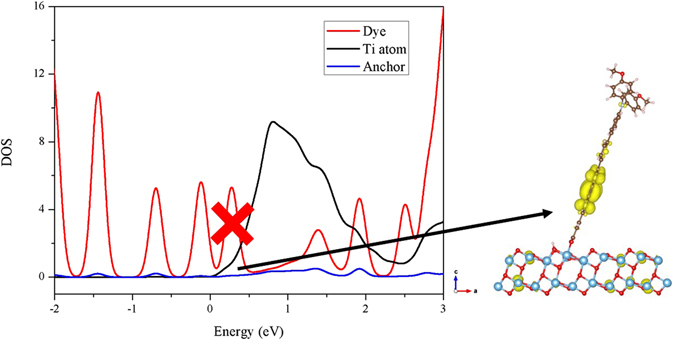
The PDOS along with its partial charge density for the peak at conduction band minimum of dye Dπ-CDM-COOH adsorbed on TiO_2_ surface; isovalue 0.0004 a.u.

The detailed information of the electronic features of adsorbed designed full dyes on the surface can be obtained from the density of states (DOS) analysis. The plotted DOS for the designed dyes are shown in Fig. 9. The Fermi levels of the studied systems in DOS figures are adjusted to zero. As can be seen from DOS, the withdrawing ability of different acceptor moieties influenced the band gap. We found that the strongest acceptor (CDM) has a minimum gap and weakest acceptor CDT has a maximum gap. The electronic coupling between the dyes and oxide surface can be obtained by integrating the overlap area of anchoring group and surface Ti atoms in the DOS plot, and the values are listed in Table 6. It has been observed that the presence of cyano group decreases the electronic coupling between the dyes and surface, thereby decrease in electron injection ability. Also, the substitution of different acceptors does not have a significant influence on the electronic coupling between dyes and surfaces. However, the strength of electron withdrawing ability of acceptors plays an important role in improving the optoelectronic properties of the dyes^14, 17^. Further, to study the effect of different acceptors on the electron injection ability, we plotted the PDOS for different acceptors substituted full dyes. In addition, the partial charge density distribution for the peak at conduction band minimum in the PDOS are obtained. The PDOS and partial charge density distribution plots for Dπ-CDM-COOH are given in Fig. 10 and for Dπ-TP-COOH and Dπ-CDT-COOH are given in Figures S7 and S8 of supporting information. As can be seen from partial charge density distribution plot in Fig. 10, the electrons are mainly localized in acceptor moiety rather than the anchoring group, which leads to dropping in electron injection to the surface. This is in agreement with our earlier findings^17^, that LUMO of the dye with the strongest acceptor (CDM) localized mainly on the acceptor moiety, not in the anchoring group. However, for the short wavelength regions, we noticed significant coupling between the anchoring group and surface atoms. In the case of other two acceptors, (TP and CDT) we found the noticeable coupling between anchoring group and surface atoms for all the three bands. Further, the LUMO of the dye with the strongest acceptor (CDM) is lower than the conduction band of TiO_2_, which reduces the electron injection ability. Hence, the strength of the acceptor moiety is important in the dye sensitizer; it should not be either too strong or too weak in order to be an efficient dye.

**Table 6:** The calculated integral area between anchoring group and two surface Ti atoms.

System	COOH
Dπ-CDM	0.500
Dπ-^CN^CDM	0.403
Dπ-TP	0.453
Dπ-^CN^TP	0.399
Dπ-CDT	0.454
Dπ-^CN^CDT	0.387

**Figure 11:**
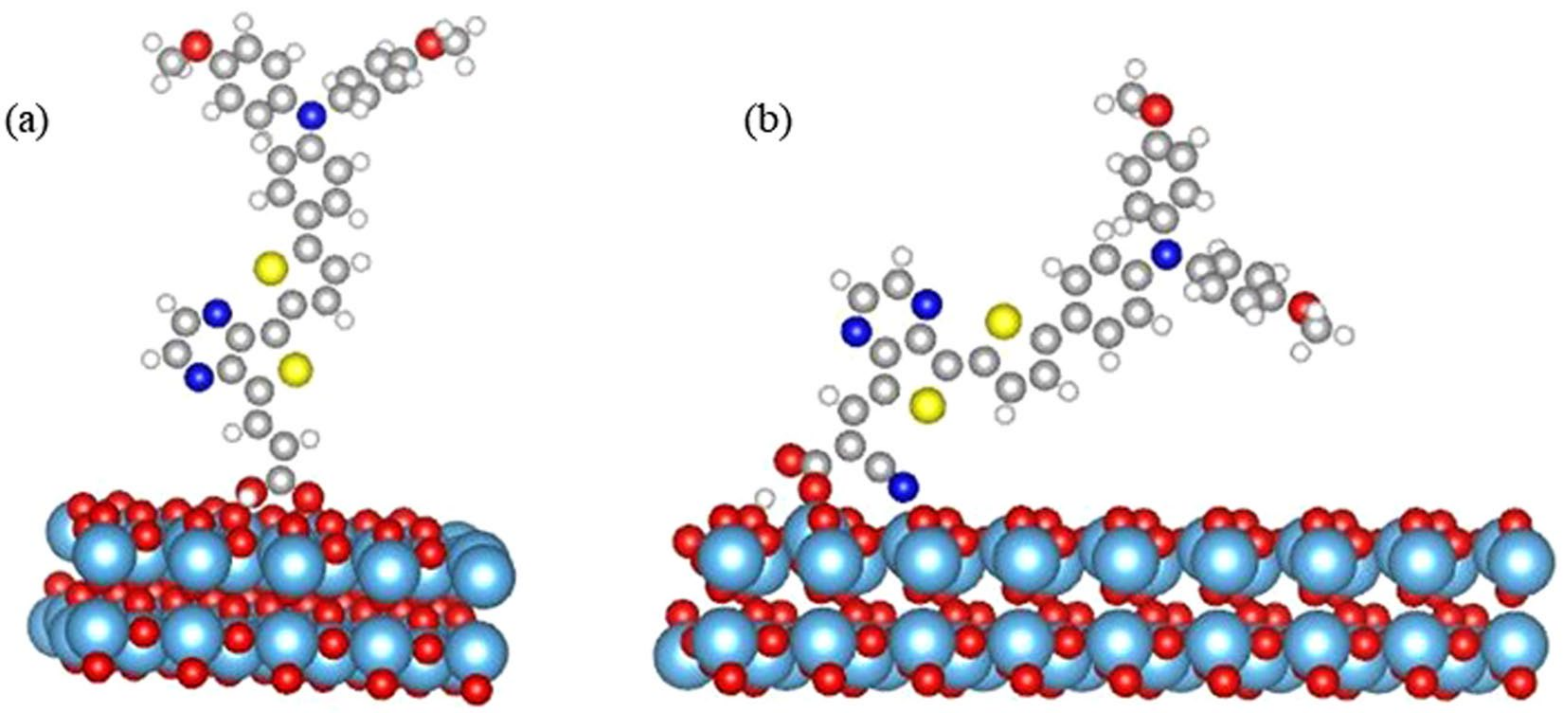
The optimized structures of (**a**) Dπ-TP-COOH dye in A3 configuration and (**b**) Dπ TP-^CN^COOH dye in B5 configuration on TiO_2_ anatase (101) surface.

It is well known that the PCE of DSSCs also affected by the dye loading concentration. Recently, Zhang *et al*.^58^ reported the lower PCE values in D-D-π-A type of dyes because of the reduced dye loading. Also, Ooyama *et al*.^59^, found that the substitution of two pyridyl groups as an anchoring group increased the dye loading. Hence it is important to investigate the effect of cyano group on the dye loading of this designed dyes. For this, we considered two most important configurations based on our model dyes results; one is the most stable B5 configuration, and another one is most favorable A3 configuration. We have extended the supercell to 1 × 8 to adsorb the B5 configuration on the surface. To minimize the computational time, we have considered only six atomic layers for B5 configurations and top three layers and dye were relaxed during the calculation. The optimized structures for both A3 and B5 configurations are shown in Fig. 11. As we can see from this fig, the dye in A3 configuration adsorbed perpendicular to the surface, whereas, dye with B5 configuration adsorbed almost parallel to the surface indicating that the dye adsorbed via A3 configuration on the surface has larger dye loading than B5 configuration.

In summary, the preferred adsorption configurations of the designed model dyes on anatase TiO_2_ (101) surface were determined using first principles based calculations. We have considered monodentate ester-type (MET), bidentate chelating (BC) and bidentate bridging (BB) configurations and investigated their adsorption properties using van der Waals corrected DFT methods. Also, we present a detailed investigation of the effects of the presence of cyano group in the anchoring moiety of designed organic dyes on both adsorption stability and photovoltaic properties such as open circuit voltage and electron injection ability. We find that the inclusion of cyano group increases the adsorption energy only when it adsorbs via CN with the surface. The presence of cyano group in the anchoring group decreases the electron injection ability when it does not involve in the binding with surface atoms, whereas it has very minimum contribution when it binds with surface atoms. Our calculated results indicate that the bidentate bridging configuration A3 is most appropriate adsorption mode for the dyes on the surface because of its higher electron injection ability, larger *V*_*OC*_ values and higher dye loading. Based on our results, we suggest that it is possible to replace the carboxylic hydrogen atom by sodium salt (-COONa) in the A3 (BB) configurations that can further increase adsorption stability and its photovoltaic properties. We hope that this theoretical work will guide experimental studies directed towards the practical implementation of these designed dyes with most favored adsorption configuration for the fabrication of DSSC device.

### Computational Details

The interaction of dye/TiO_2_ systems has been studied using density functional theory (DFT) framework in a plane-wave basis implemented in Vienna Ab-initio simulation package (VASP 5.4)^60, 61^. The exchange-correlation energy was calculated within the generalized gradient approximation (GGA) using Perdew-Burke-Ernzerhof (PBE) functional^62^. The plane wave cutoff energy was set to 400 eV, and 10 × 10 × 4 and 2 × 3 × 1 k-point mesh with Monkhorst-Pack scheme were used for bulk and surface of anatase TiO_2_ calculations, respectively. It is well known that the photovoltaic properties of DSSCs strongly depend on the orientations or reconstructions of the semiconductor surface^63, 64^. Earlier studies demonstrate that anatase phase of TiO_2_ dominates the rutile phase regarding electron transport, short-circuit photocurrent, photocatalytic activity, and dye loading^65, 66^. Also, many studies have reported that first crystalline phase formed during TiO_2_ synthesis is anatase^67, 68^. Moreover, the formation of anatase phase requires relatively less temperature (<150 °C) than rutile phase and thus it has attracted greater interest in solar cells^69^. Considering all these merits, anatase TiO_2_ phase was selected for the investigation of dye adsorption characteristics. The bulk lattice parameters were calculated by varying the ion position, cell shape, and volume throughout minimization. The threshold of SCF convergence was set to 10^−5^ eV and 10^−3^ eV/Å for the total energy and force on each atom, respectively. The calculated lattice parameters are a = b = 3.8005 Å, c = 9.4887 Å which are similar to experimental values (a = b = 3.7848 Å, c = 9.5124 Å).

The TiO_2_ anatase (101) surface was modeled by two molecular layers, which contains 64 Ti and 128 O atoms. Here, we considered the most stable surface based on their surface energy benchmark calculations and a 2 × 4 supercell was used to study the adsorption of our designed metal free dyes. In all calculations, four bottom atomic layers are fixed, while the other layers and dyes in the supercell are allowed to relax. The vacuum gap of 40 Å is used to avoid the interaction of dyes with the adjacent slab. It has confirmed in earlier studies that the inclusion of van der Waals (vdW) functional along with GGA improved the energies of adsorbed molecules on the oxide surfaces^70, 71^. Hence, we used optB86b-vdW^72, 73^ functional to treat the long-range interactions of the studied system.

## Electronic supplementary material

supporting informaiton
