# Supplementary material for: Revealing the influence of Cyano in Anchoring Groups of Organic Dyes on Adsorption Stability and Photovoltaic Properties for Dye-Sensitized Solar Cells
Source: Sci Rep. 2017 Jul 10;7:4979. doi: 10.1038/s41598-017-05408-8 (PMC5504066; doi:10.1038/s41598-017-05408-8)
Supplement: Supplementary file 1 — supporting informaiton [file 41598_2017_5408_MOESM1_ESM.pdf]

Supporting information for

Revealing the influence of Cyano in Anchoring Group of Organic Dyes on Adsorption Stability and Photovoltaic Properties for Dye Sensitized Solar Cells

Wei-Chieh Chen, Santhanamoorthi Nachimuthu* and Jyh-Chiang Jiang*

Department of Chemical Engineering, National Taiwan University of Science and Technology, Taipei 106, Taiwan, R.O.C.

* Corresponding authors : jcjiang@mail.ntust.edu.tw (JCJ), santhanamoorthi@gmail.com (SN)
Telephone: +886-2-27376653. Fax: +886-2-27376644.

Table S1. The calculated adsorption energies (eV) for the dyes with different lengths adsorbed on TiO_2 anatase (101) surface.

**Dyes**	**E_{ads}(in eV)**
**An**	-0.43
**A-An**	-0.44
**π-A-An**	-0.47
**D-π-A-An**	-0.48

Table S2. The calculated adsorption energies (E_{ads}, in eV) and selected bond distances (d, in Å) and bond lengths (r, in Å) of all the studied configurations at PBE functional (See Figure 1 for configurations)

Parameters	I		II		III		IV		V	
	I_A	I_B	II_A	II_B	III_A	III_B	IV_A	IV_B	V_A	V_B
E_{ads} (PBE)	-0.85	-0.76	-0.82	-0.69	-0.20	-0.25	-0.45	-0.40	0.24	0.28
$r_{O\text{-}TiN}$ (N = 1 or 2)	($O\text{-}Ti_2$)<br>2.131	($O\text{-}Ti_2$)<br>2.178	($O\text{-}Ti_2$)<br>2.172	($O\text{-}Ti_2$)<br>2.210	($O\text{-}Ti_1/Ti_2$)<br>1.936<br>2.668	($O\text{-}Ti_1/Ti_2$)<br>1.968<br>2.664	($O\text{-}Ti_1/Ti_2$)<br>2.057<br>2.078	($O\text{-}Ti_1/Ti_2$)<br>2.111<br>2.086	($O\text{-}Ti_2$)<br>2.086<br>2.098	($O\text{-}Ti_2$)<br>2.123<br>2.106
$d_{H...ON}$/ $r_{H\text{-}ON}$ (N = 1-5)	($H\text{-}O_4$)<br>1.675	($H\text{-}O_4$)<br>1.660	($H\text{-}O_2$)<br>2.018	($H\text{-}O_2$)<br>2.078	($H\text{-}O_1$)<br>0.972	($H\text{-}O_1$)<br>0.972	($H\text{-}O_1$)<br>0.972	($H\text{-}O_1$)<br>0.973	($H\text{-}O_2$)<br>0.973	($H\text{-}O_2$)<br>0.973
$d_{O...H}/r_{OH}$	1.013	1.020	0.994	0.994	2.543	2.536	2.476	2.516	3.135	3.149

Modes	VI		VII		VIII		IX		
	VI_A	VI_B	VII_A	VII_B	VIII_A	VIII_B	IX_A	IX_B	
E_{ads} (PBE)	0.01	-0.32	-0.57	-0.75	-0.70	-0.65	0.40	-0.13	
$r_{O\text{-}Ti5c}$	($O\text{-}Ti_1$)<br>2.582	($O\text{-}Ti_1$)<br>2.798	($O\text{-}Ti_1$)<br>2.250	($O\text{-}Ti_1$)<br>2.369	($O\text{-}Ti_1$)<br>2.098	($O\text{-}Ti_1$)<br>2.009	($O\text{-}Ti_1$)<br>1.873	($O\text{-}Ti_1$)<br>1.975	
$d_{H...ON}$/ $r_{H\text{-}ON}$ (N = 1-5)	($H\text{-}O_1$)<br>2.350	($H\text{-}O_1$)<br>2.294	($H\text{-}O_3$)<br>3.304	($H\text{-}O_3$)<br>3.328	($H\text{-}O_3$)<br>1.682	($H\text{-}O_3$)<br>1.025	($H\text{-}O_5$)<br>0.987	($H\text{-}O_5$)<br>0.986	-
$d_{O...H}/r_{OH}$	0.980	0.982	0.980	0.982	1.014	1.679	2.718	2.919	
$r_{N\text{-}TiN}$ (N = 1 or 2)	-	($N\text{-}Ti_2$)<br>2.375	-	($N\text{-}Ti_2$)<br>2.350	-	($N\text{-}Ti_2$)<br>2.289	-	($N\text{-}Ti_2$)<br>2.276	

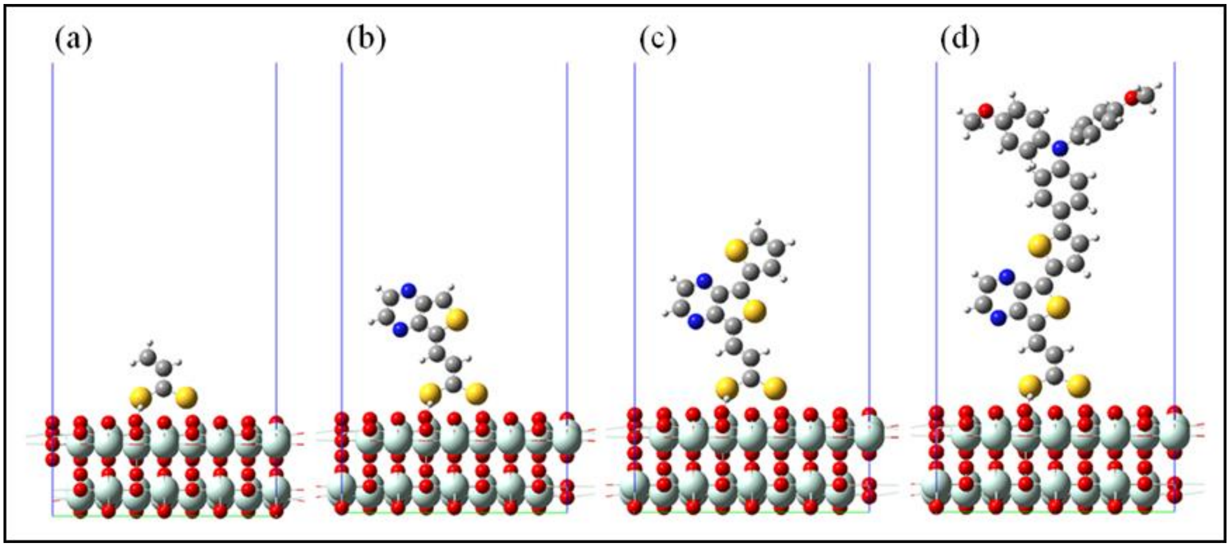

Figure S1. The optimized geometries of model dye with different lengths (a) An, (b) A-An, (c) π-A-An, and (d) D-π-A-An on adsorbed on TiO_2 surface. (An-Anchoring group, A-Acceptor, π-π Bridge, D-Donor)

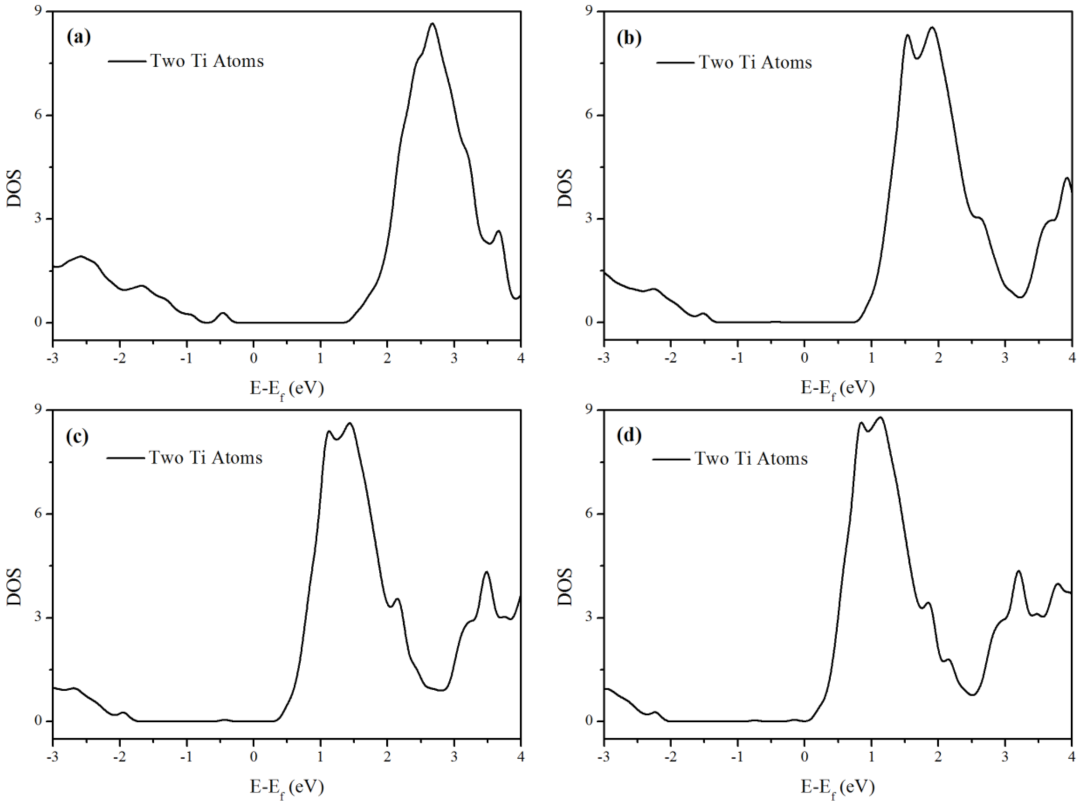

Figure S2. The density of state of two surface Ti atoms after the adsorption of model dyes with different lengths ((a) An, (b) A-An, (c) π-A-An, and (d) D-π-A-An, An-Anchoring group, A-Acceptor, π-π Bridge, D-Donor)

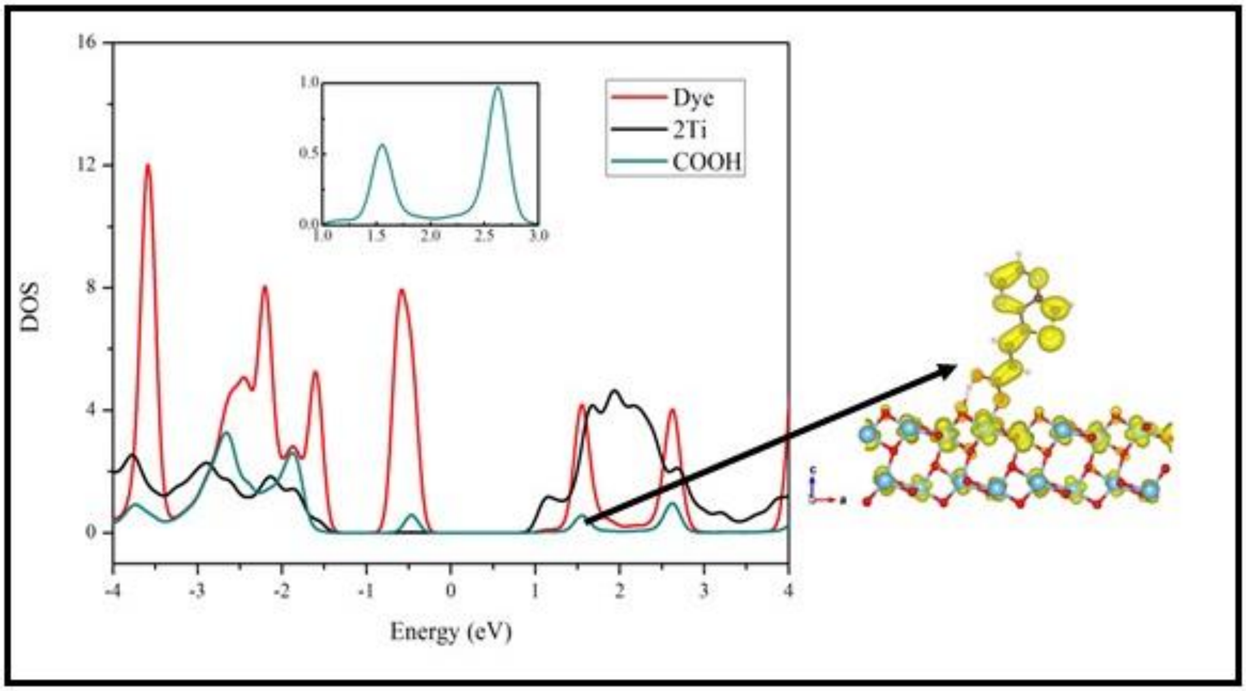

Figure S3. The DOS plot for the model dye adsorption configuration A2 along with its partial charge density for the band corresponds to long wavelength absorption band at 0.0004 isovalue.

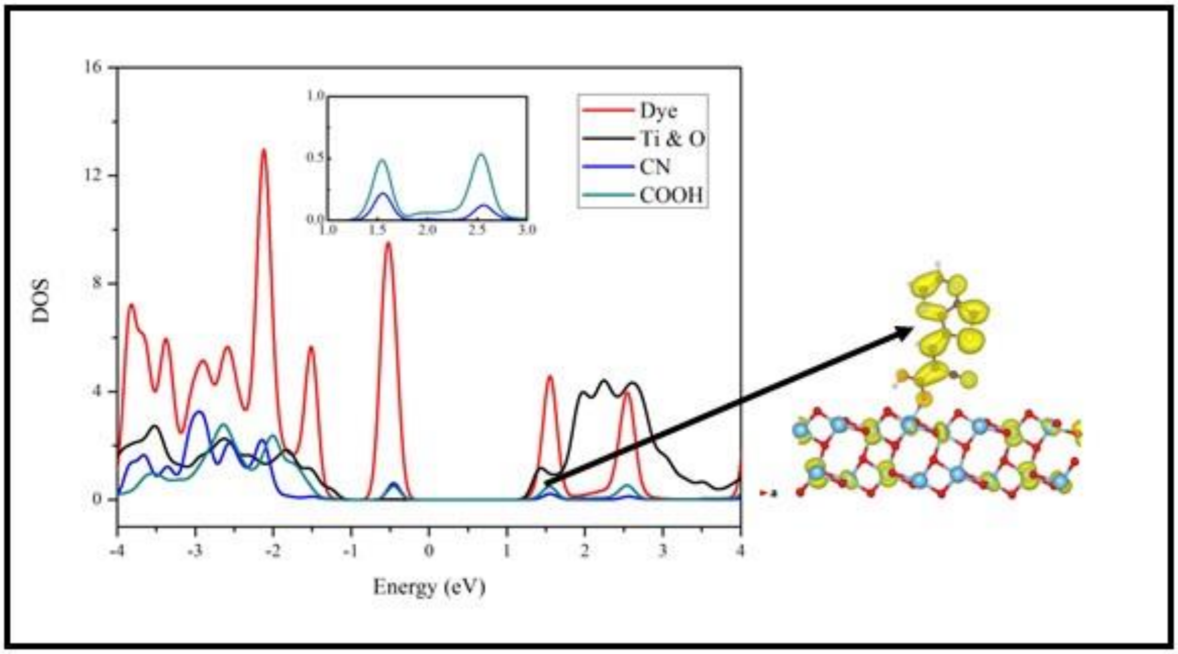

Figure S4. The DOS plot for the model dye adsorption configuration B2 along with its partial charge density for the band corresponds to long wavelength absorption band at 0.0004 isovalue.

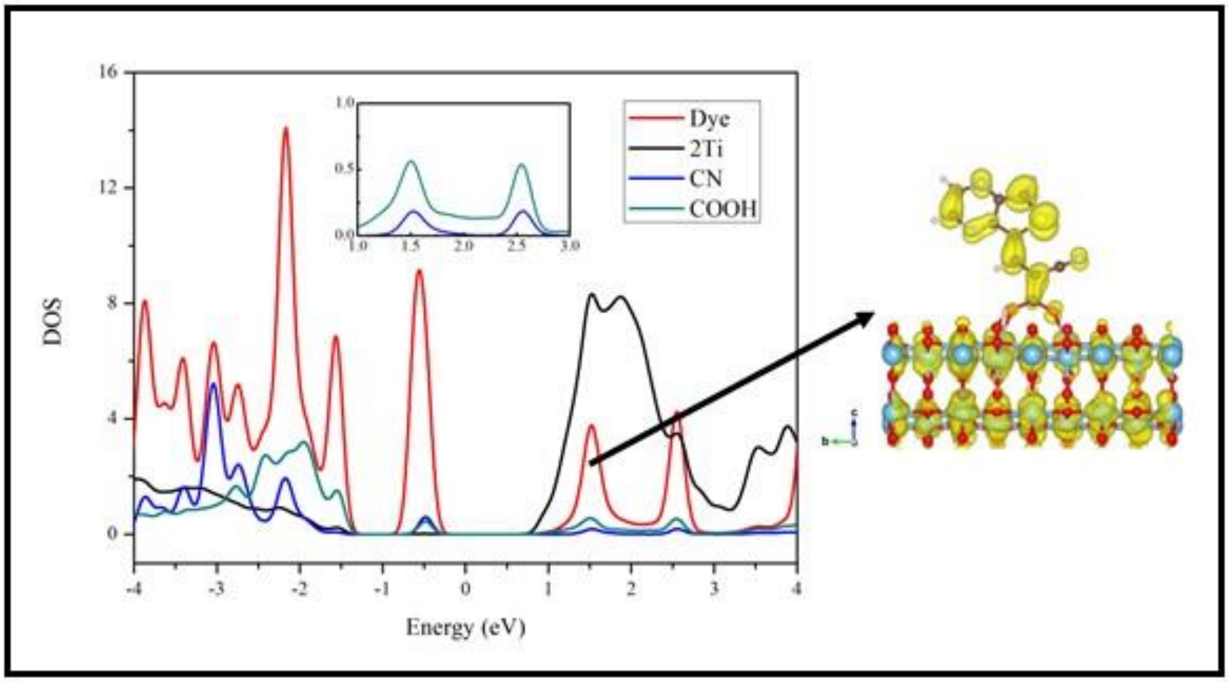

Figure S5. The DOS plot for the model dye adsorption configuration B3 along with its partial charge density for the band corresponds to long wavelength absorption band at 0.0004 isovalue.

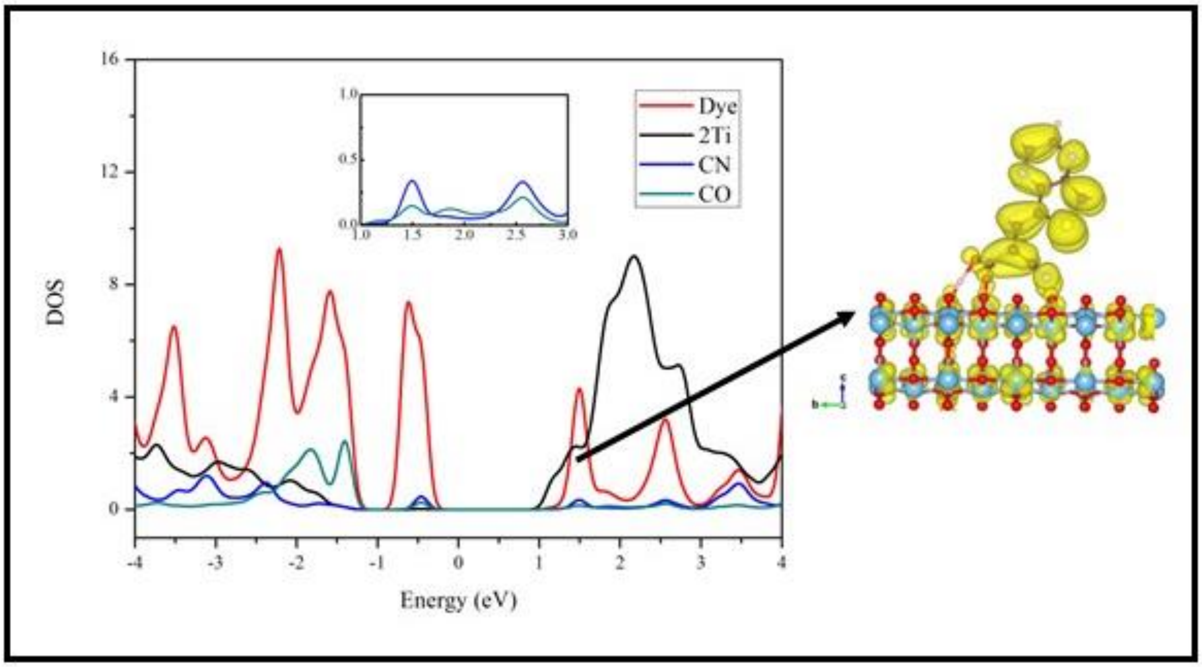

Figure S6. The DOS plot for the model dye adsorption configuration B5 along with its partial charge density for the band corresponds to long wavelength absorption band at 0.0004 isovalue.

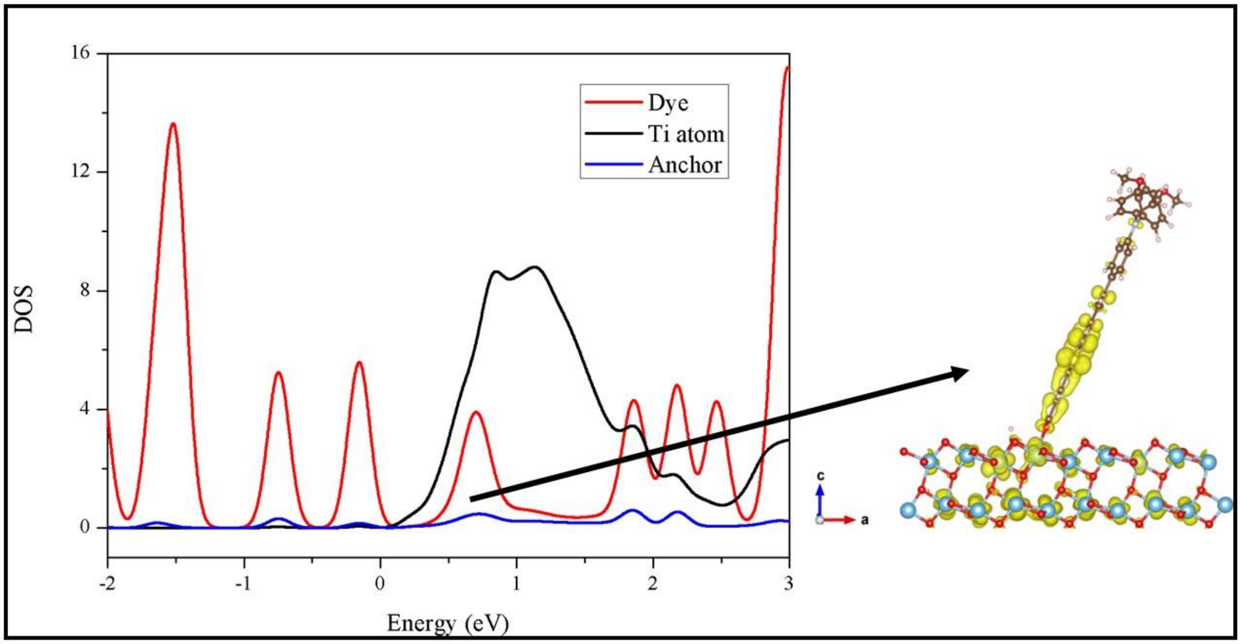

Figure S7. The PDOS along with its partial charge density for the peak at conduction band minimum of dye Dπ-TP-COOH adsorbed on TiO_2 surface; isovalue 0.0004 a.u.

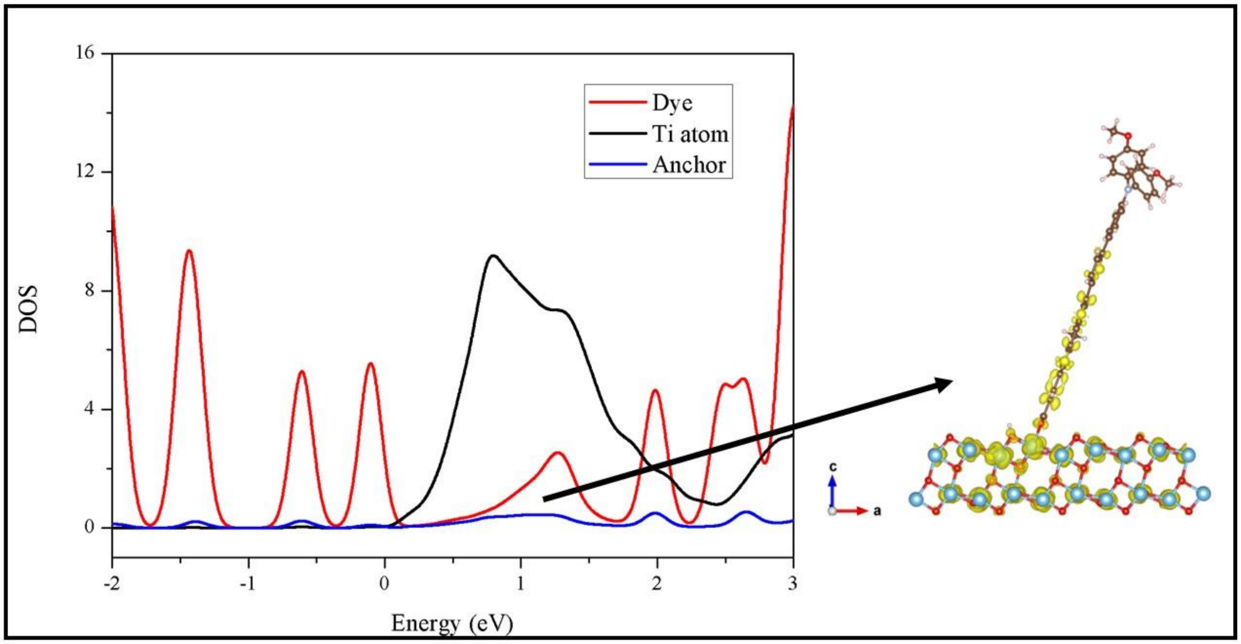

Figure S8. The PDOS along with its partial charge density for the peak at conduction band minimum of dye Dπ-CDT-COOH adsorbed on TiO_2 surface; isovalue 0.0004 a.u.
